# A novel method for extracting circulating cell‐free DNA from whole blood samples and its utility in the non‐invasive prenatal test

**DOI:** 10.1002/pd.6212

**Published:** 2022-08-05

**Authors:** Hongbin Zhong, Liuhong Zeng, Mengyuan Tao, Yuchen Ye, Yanqi Wang, Lei Hou, Miaofeng Wu, Hui Liu, Hongyun Zhang, Meifang Tang

**Affiliations:** ^1^ Clinical Laboratory of BGI Health BGI‐Shenzhen Shenzhen China; ^2^ BGI Education Center University of Chinese Academy of Sciences Shenzhen China

## Abstract

**Objective:**

We verified a magnetic bead‐based, simple, and fast method for circulating cell‐free DNA (cfDNA) extraction from whole blood samples(CEWB) and characterised its utility in non‐invasive prenatal testing (NIPT).

**Method:**

We extracted cfDNA from both plasma and whole blood of the patients using CEWB and compared it to that extracted using a Qiagen extraction kit; droplet digital polymerase chain reaction test was used to calculate the fragment size bias. In all, 304 samples were used for NIPT.

**Results:**

The CEWB group (mean ± standard deviation [SD]: 4.34 ± 0.41 ng/ml plasma) reported less DNA weight yield than the Qiagen group (4.90 ± 0.50 ng/ml plasma). There was no significant difference between the CEWB group and the Qiagen group in the gene fragments (136 bp: *p* = 0.064 and 420 bp: *p* = 0.534). In a parallel cohort study to characterise the utility of the CEWB method in NIPT, the treatment group extracted by CEWB showed a sensitivity of 100%, a specificity of 99.65%, and a positive predictive value of 95%.

**Conclusions:**

This study demonstrated that CEWB achieves an acceptable yield of DNA without contamination from genomic DNA. Subsequent clinical experiments in a parallel cohort indicated its utility for NIPT.

## Key points

**What's already known about this topic?**

- Extraction of cell‐free DNA (cfDNA) from blood or plasma for non‐invasive prenatal testing is currently the standard method in use, but the centrifugation and transfer of supernatant required by this method is cumbersome, difficult, time‐consuming, and costly.

**What does this study add?**

- In our method, the cfDNA is directly extracted from blood, simply by adding the blood to magnetic beads for cfDNA extraction. Performance validation of the method was done to determine method reliability.

## 1 INTRODUCTION

Circulating cell‐free DNA (cfDNA) was discovered in human blood in 1948^1^; foetal DNA in maternal plasma and serum was discovered in 1997.^2^ Since then, cfDNA has become an important method of liquid biopsy, and it is used as a non‐invasive screening tool for many diseases, especially solid tumours, and foetal genetic abnormalities.^3^, ^4^, ^5^, ^6^, ^7^ Non‐invasive prenatal testing (NIPT) uses next‐generation sequencing (NGS) technologies using cell‐free foetal DNA from maternal plasma to detect certain genetic conditions such as trisomy (T) 21, T18, and T13 during pregnancy. It has a sensitivity and specificity (approximately 99%). Since 2011, massively parallel screening for foetal aneuploidies has become available in more than 60 countries. Increasing use of the cfDNA‐based NIPT has created unprecedented challenges in automation in the biotechnology industry.^5^ Current cfDNA extraction methods^8^, ^9^ mainly include: (I) column‐based methods, such as the QIAamp circulating nucleic acid kit^10^, ^11^, ^12^, ^13^, ^14^; (II) magnetic bead‐based methods, such as the NextPrep‐Mag™ cfDNA isolation kit^13^; (III) polymer‐mediated enrichment, such as the PME free‐circulating DNA extraction kit.^13^ However, the pre‐processing separation of serum or plasma from whole blood (WB) samples limits the performance of the above cfDNA extraction methods. Pre‐processing is a time‐consuming method that requires high‐speed centrifugation at low or room temperature for 15–30 min, while avoiding contamination of the blood cells during pipetting operations.^2^, ^9^, ^14^ The main challenge faced by many engineers is the automation of the separation of serum or plasma cost‐effectively. This includes intelligent control of the centrifuge for automatic tube insertion, removal, identification of plasma and buffy coat, shorter (<30 min) separation operations, reduced cost, and increased throughput stability. Isolating cfDNA from peripheral blood instead of plasma or serum is not often attempted. Pandoh et al. reported a high‐throughput protocol for isolating tumour cfDNA,^15^ but this is complex and requires a long incubation time (>1 h). Here, we introduce a magnetic bead‐based, simple, and fast cfDNA extraction method from WB samples, which we have termed CEWB; additionally, we estimate its effect on DNA yield and fragment size bias and characterise its utility for NIPT. This study is one of the first investigations on cfDNA extraction from WB samples. We used Cell storage solutions, which have trace amounts of formaldehyde released from imidazolidinyl urea which coagulates the proteins, immobilises the cells, and prevents them from rupture and genomic DNA (gDNA) release. The cfDNA is then directly bound to blood outside the blood cells by electrostatic force using amino magnetic beads, without the need for a high salt environment as carboxylated magnetic beads and other substances (red blood cells, white blood cells, etc.) are removed by wash buffer. Subsequently, cfDNA is eluted off by the elution buffer in the kit and purified, and fragments are selected using carboxyl magnetic beads to remove impurities and increase the proportion of target cfDNA (e.g., foetal or tumour cfDNA).

## 2 METHODS

### 2.1 Participants

A total of 366 pregnant women with a gestation period between 12 and 24 weeks were enrolled from 2020 to 2021. We confirmed 20 NIPT‐positive samples with foetal T21, T18, or T13 by karyotyping and/or chromosomal microarray analysis (CMA). A total of 282 NIPT‐negative samples were followed up 3 months after delivery and were found to be true negative. All participants provided written informed consent before blood collection, and the study was approved by the Institutional Review Board (IRB) of the BGI (NO. BGI‐IRB 21008). All WB samples collected in EDTA tubes were used for plasma isolation or cfDNA extraction using CEWB within 8 h.

We used the following exclusion criteria: a gestation period of <12 + 0 weeks; one spouse with a chromosomal abnormality; treatment for an abnormality, such as stem cell therapy (within 1 year) or exogenous DNA treatment (within 4 weeks); a foetal ultrasound indicating structural abnormalities; a family history of genetic disorders or a high risk of genetic disorders in the foetus; a combination of malignant tumours during pregnancy (except for benign uterine myoma); or a multiple pregnancy.

### 2.2 CfDNA extraction from WB samples

The cfDNA was extracted from WB samples by modifying the magnetic bead extraction kit‐Whole Blood Cell‐Free DNA Extraction Kit (cat #CFDNAWBB50, Jiashan Zhijian Tech Co., Ltd., Zhejiang, China). There are two types of magnetic beads in the kit. Type 1 beads are amino magnetic beads which have a superparamagnetic silica matrix and an active amino group. These beads can bind to DNA based on electrostatic force without a high salt environment and can bind to free DNA in blood directly when added to blood. Type 2 magnetic beads are carboxyl magnetic beads which have a superparamagnetic silica matrix and an active carboxyl group. When the outer surface of the magnetic beads with carboxyl functional group is modified in the purification buffer system containing polyethylene glycol (PEG), high salt ions, etc., the DNA is adsorbed by forming an ionic DNA‐salt ions‐carboxyl bridge. This binding is reversible; the ionic bridge is dissolved in TE (Tris‐EDTA) buffer without PEG and salt ions to obtain purified DNA. This is the type commonly used for DNA purification and can also be used for fragment selection.

**FIGURE 1:**
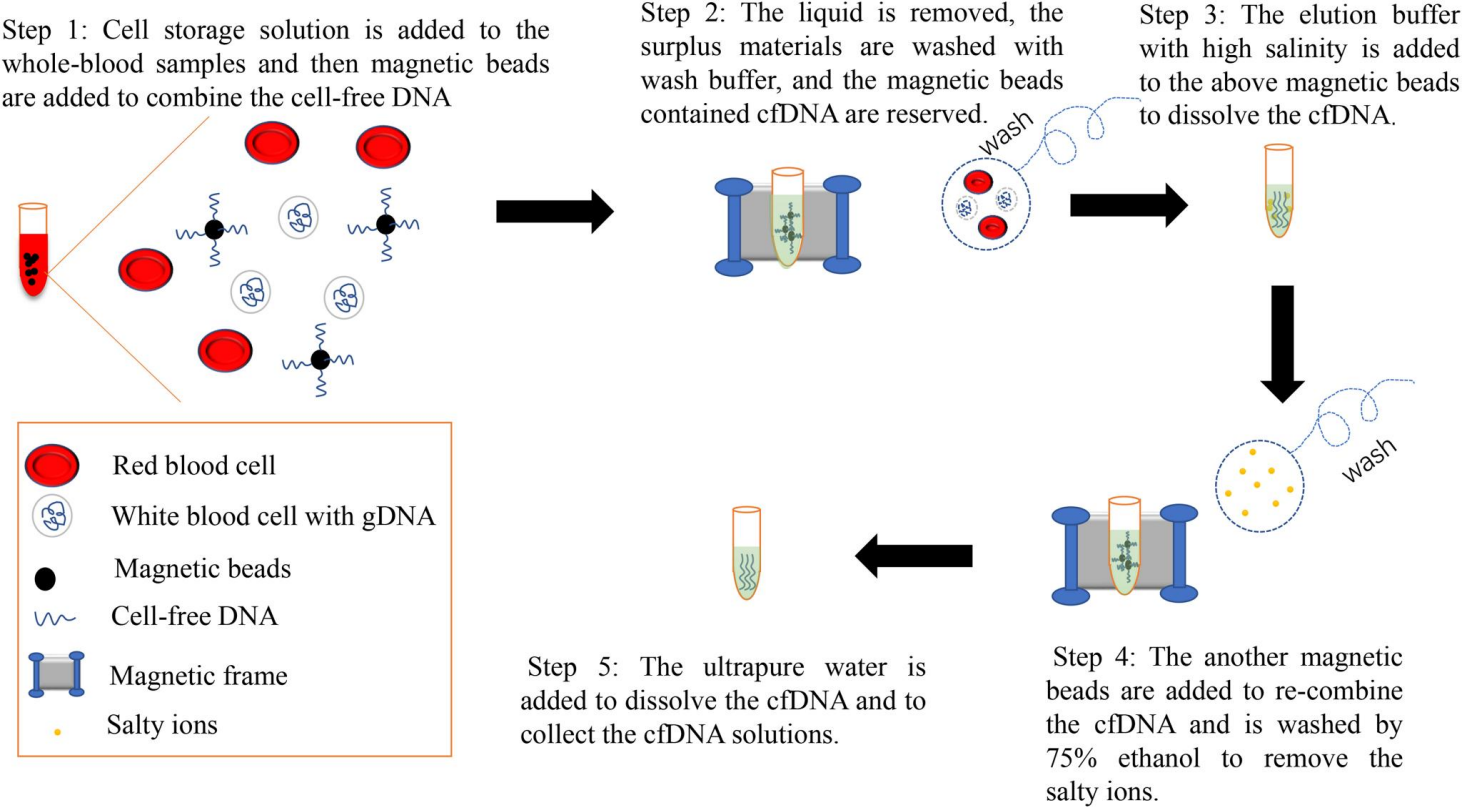
The working principle of CEWB. CEWB, cell‐free DNA (cfDNA) extraction from whole blood samples; cfDNA, circulating cell‐free DNA

The two types of magnetic beads were prepared and incubated for 10 min at room temperature. Type 1 magnetic beads (30 μl) were mixed with 100 μl wash buffer from the kit, the supernatant was discarded, and then 30 μl wash buffer was re‐added; 0.1× volume of Cell storage solution (the main ingredient is imidazolidinyl urea) from the kit was added to 1 ml WB before tapping and mixing (not required for plasma samples). The blood sample was mixed with the type 1 magnetic beads, incubated at room temperature for 5 min, centrifuged at low speed for 5 s, placed on a magnet for 2 min, then the blood was discarded. The sample was then mixed with 500 μl wash buffer, centrifuged at low speed for 5 s, placed on a magnet for 30 s, and the supernatant was discarded. This procedure was repeated twice. We then mixed 40 μl of elution buffer for 4 min and aspirated 40 μl of the supernatant. The above supernatant was mixed with 50 μl type 2 magnetic beads, incubated at room temperature for 5 min, centrifuged at low speed for 5 s, placed on a magnet for 5 min, supernatant was discarded, and for cfDNA fragment size enrichment and selection in the NIPT experiments, the supernatant was mixed with 20 μl type 2 magnetic beads, incubated at room temperature for 5 min, centrifuged at low speed for 5 s, placed on a magnet for 5 min, and the supernatant was aspirated into a new 1.5 ml centrifuge tube, mixed with 30 μl type 2 magnetic beads, incubated at room temperature for 5 min, centrifuged at low speed for 5 s, placed on a magnet for 5 min, and the supernatant discarded. The type 2 magnetic beads were cleaned twice with 500 μl of 75% ethanol for 5 min after the ethanol evaporated. The ultrapure water or TE buffer (≥20 μl) was mixed for 4 min, and the supernatant was aspirated into a new 1.5 ml centrifuge tube. Figure 1 illustrates the working principle of the cfDNA extraction method for WB samples.

### 2.3 CfDNA extraction from plasma samples

We used two cfDNA extraction methods for plasma samples. Plasma samples were first obtained by high‐speed centrifugation of 1 ml WB samples,^16^ and then cfDNA was extracted using either a column‐based method on a QIAamp circulating nucleic acid kit (cat #55114, Qiagen, Hilden, Germany) or a magnetic bead‐based method on a MGIEasy Circulating DNA Isolation Kit (MGI Tech Co., Ltd, Shenzhen, China). Both kits were used following the manufacturer's instructions.

### 2.4 Calculation of the DNA weight yield

cfDNA concentration was measured using a Qubit dsDNA HS Assay Kit (cat# Q32854, Thermo Fisher Scientific Inc., Waltham, MA, USA), and DNA weight was calculated as concentration × volume.

### 2.5 Preparation of simulated samples containing tumour reference material

gDNA was extracted from NCI‐H1975, a human lung adenocarcinoma cell line with a 77.7% mutation rate for EGFR‐T790M (ATCC, Maryland, USA). gDNA extracted from immortalised cell lines (originally donated by healthy individuals), was ultrasonically fragmented using a Coviras LE220 (Agilent, Santa Clara, CA, USA) under the following parameters: 40 cycles at peak power: 500, duty factor: 21, and burst: 500; and then measured using an Agilent 2100 bioanalyzer. Next, the fragmented tumour DNAs were proportionally prepared and mixed into two groups: a 5% EGFR‐T790M group and a 0.5% EGFR‐T790M group. Both groups were validated with droplet digital polymerase chain reaction (ddPCR).

The 5% EGFR‐T790M WB samples consisted of 500 μl WB from a healthy pregnant woman and 45 ng tumour DNA with 5% EGFR‐T790M. The 0.5% EGFR‐T790M WB samples consisted of 500 μl WB from a healthy pregnant woman and 90 ng tumour DNA with 0.5% EGFR‐T790M (extremely low DNA mass may result in an extremely low copy number of mutant DNA fragments after extraction and may affect the accuracy of ddPCR quantification). For the plasma samples, the 5% EGFR‐T790M samples consisted of 250 μl plasma from a healthy pregnant woman and 45 ng tumour DNA with 5% EGFR‐T790M, and the 0.5% EGFR‐T790M samples consisted of 250 μl plasma from the same healthy pregnant woman and 90 ng tumour DNA with 0.5% EGFR‐T790M.

### 2.6 The ddPCR assays

The primers and probes for the short β‐actin gene fragment (136 bp) and longer β‐actin fragment (420 bp) have been previously recorded in the literature.^16^ The following primers were used: For EGFR‐T790M: T790M‐F (5′‐ GCCTGCTGGGCATCTG‐3′), T790M‐R (5′‐ TCTTTGTGTTCCCGGACATAGTC‐3′), T790M‐P‐WildType (5′‐6‐FAM‐ATGAGCTGCGTGATGAG‐3′‐BHQ1), and T790M‐P‐MutType (5′‐HEX‐ATGAGCTGCATGATGAG‐3′‐BHQ1). The above primers and probes were synthesised by Sangon Biotech (Shanghai) Co., Ltd, Shanghai, China. The ddPCR assays were performed on the QX200TM ddPCR system (Bio‐Rad, Hercules, CA, USA) with ddPCR supermix for probes (no dUTP) kits following the manufacturer's instructions. The 22 μl PCR of the short and long β‐actin fragments were composed of 11 μl 2 × ddPCR premix supermix, 900 nM primers/250 nM of each probe, 5–7 μl DNA, and enzyme‐free water. The PCR conditions were as follows: pre‐denaturation at 98°C for 10 min, followed by 40 cycles: 95°C for 30 s, 54°C for 30 s, 72°C for 30 s, and a final step at 72°C for 10 min and 98°C for 10 min, before storage at 4°C. The EGFR‐T790M reactions were composed of 11 μl 2 × ddPCR master mix Supermix, 900 nM primers/250 nM of each probe, 10 ng DNA, and enzyme‐free water. The PCR conditions were as follows: pre‐denaturation at 95°C for 10 min, followed by 40 cycles at 94°C for 30 s, 57°C for 1 min, and finally at 98°C for 10 min, before storage at 4°C. The droplets were subsequently produced by an automated droplet generator (Bio‐Rad, Hercules, CA, USA), 40 μl transferred, and amplified on a C1000 Touch thermal cycler with a 96–deep well reaction module. After amplification, the 96‐well plate was placed in a QX200TM droplet reader and was analysed using QuantaSoft software (Bio‐Rad, Hercules, CA, USA).

### 2.7 NIPT

The Fetal Chromosome Aneuploidy (T21, T18, T13) Assay Kit (BGI Biotechnology, Wuhan, China) which uses the combined probe anchored polymerase sequencing method was used to construct a DNA library from the cfDNA on a MGISP‐960 high‐throughput automated sample preparation system (MGI, Shenzhen, China). The cfDNA was repaired to obtain a blunt end and modified at the 3′ end to get a dATP as a sticky end. A dTTP tailed adapter sequence was ligated to both ends of the DNA fragments. The ligated fragment was then amplified for 14 cycles. PCR products were pooled, and the PCR product concentration was determined using Qubit. The PCR product was heat‐denatured together with a special molecule and the single‐strand molecule was ligated using DNA ligase, finally obtaining a single‐strand circular DNA library. Sequencing was implemented using the MGISEQ‐2000RS high‐throughput sequencing set (FCL SE50), on a MGISEQ‐2000 sequencer (MGI, Shenzhen, China). Single‐end 50 bp sequencing reads of at least 5 million per sample were produced. A binary hypothesis strategy was developed for detection, using the bioinformatics pipelines for T21, T18, and T13.^17^, ^18^ A T‐score >4 for Chr21/Chr18/Chr13 was classified as trisomy.

### 2.8 Statistical analyses

Statistical analyses were performed using Excel 2010 and R software (version 3.6.0). The boxplot, violin plot, and dot‐plot diagrams were implemented by the ‘ggplot2’ R library. The *p*‐value was calculated using the student's *t*‐test between two groups. *p* < 0.05 was considered statistically significant. The 95% confidence interval (CI) was calculated using the Wilson CI. Results are presented as mean ± standard deviation (SD) and coefficient of variation (CV).

## 3 RESULTS

### 3.1 DNA weight yield and fragment size bias

The WB samples from 22 pregnant women were divided equally into two groups. CEWB and the Qiagen kit were used to extract DNA from 1 ml WB and from plasma isolated from 1 ml WB, respectively. Subsequently, the samples from both groups were examined with Qubit to calculate the DNA weight yield, and ddPCR was used to calculate the copies per mL of WB for the 136 bp and 420 bp fragment lengths.

**FIGURE 2:**
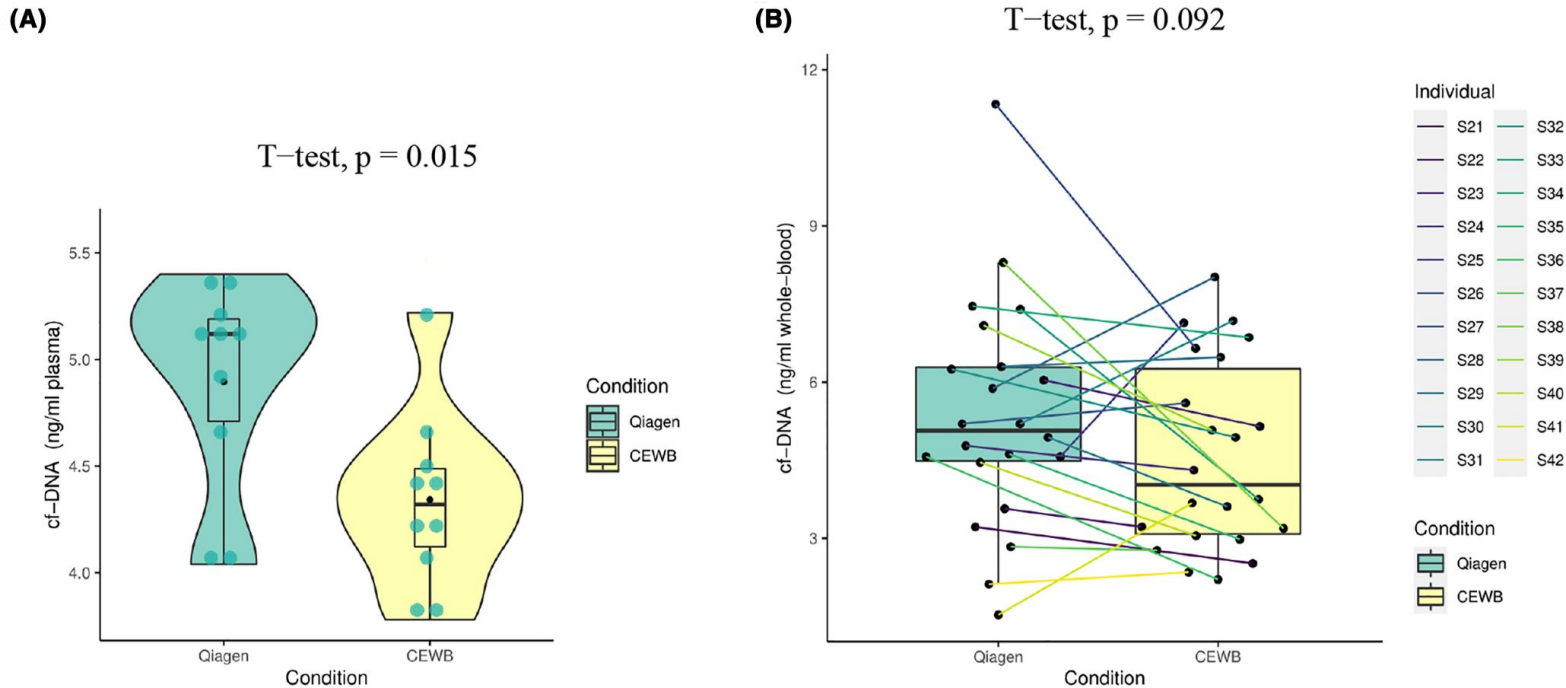
Quantitative comparison of DNA weight yield between CEWB and Qiagen kit. (A) Boxplot, violin plot, and dot‐plot diagrams of DNA weight yield obtained from mixed plasma, measured with Qubit. (B) Boxplot diagram of DNA weight yield obtained from 22 WB samples, measured with Qubit. CEWB, cell‐free DNA (cfDNA) extraction from whole blood samples; WB, whole blood

As shown in Figure 2 and Table S1, the Qiagen‐plasma group had higher yield of DNA (4.90 ± 0.50 ng/ml; 10.17%) than the CEWB‐plasma group (4.34 ± 0.41 ng/ml; 9.55%), although there was similar repeatability and stability. There was a significant difference between the two methods (*p* = 0.015).

**FIGURE 3:**
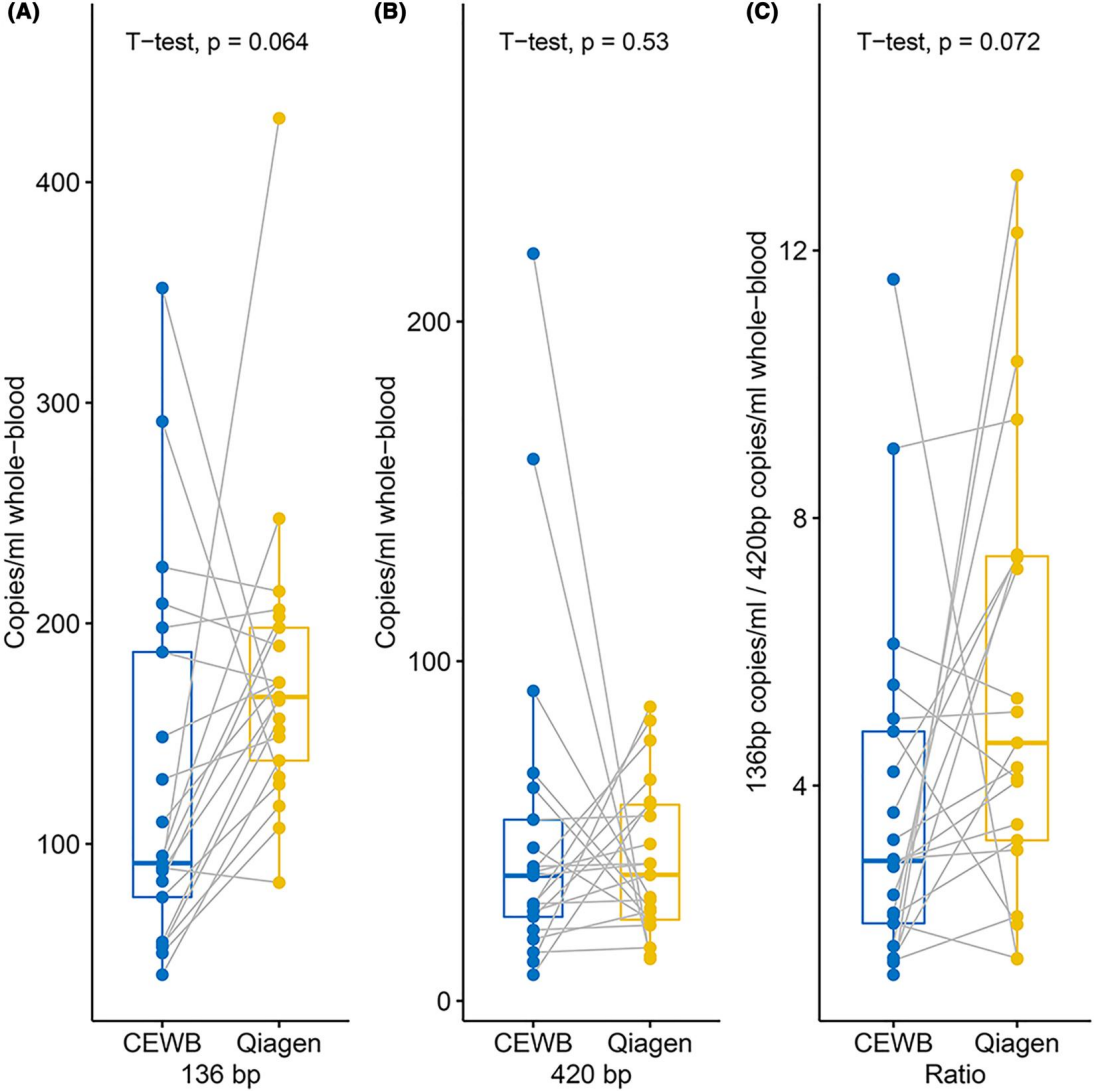
Quantitative comparison of fragment size bias between CEWB and Qiagen kit. (A) Boxplot diagram of 136 bp fragment lengths, measured with ddPCR. (B) Boxplot diagram of 420 bp fragment lengths, measured with ddPCR. (C) Boxplot diagram of 136 bp/420 bp ratio, measured with ddPCR. CEWB, cell‐free DNA (cfDNA) extraction from whole blood samples; ddPCR, droplet digital polymerase chain reaction

The copy number of the 136 bp fragment obtained by CEWB was significantly different from that obtained by Qiagen (*p* = 0.064), while the copy numbers of the 420 bp fragment were not significantly different between the two extraction methods (*p* = 0.534; Figure 3); therefore, indicating that the differences in DNA extracted by two kits were mainly due to the differences in the amount of short fragments (cfDNA) obtained.

### 3.2 DNA quantification and purity analysis from the tumour reference material

**FIGURE 4:**
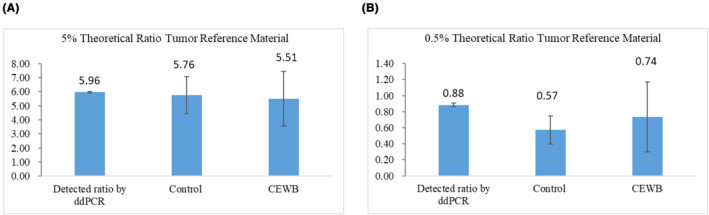
Histogram of 5% (A) and 0.5% (B) theoretical mutation from simulated samples extracted using CEWB and the magnetic bead‐based kits (control) and detected by ddPCR. CEWB, cell‐free DNA (cfDNA) extraction from whole blood samples; ddPCR, droplet digital polymerase chain reaction

The DNA of 5% or 0.5% theoretical ratio tumour reference material was mixed with the normal WB samples as simulated samples, divided equally into two portions, extracted using the CEWB method from 1 ml WB and the magnetic bead‐based kits from plasma isolated from 1 ml WB as controls, respectively. Subsequently, we measured the mutation ratio with ddPCR. Figure 4, Table S4, and Table S5 show an increase in the detected mutation ratio from the tumour reference material when compared to the theoretical ratio as well as a decrease and instability in the detected mutation ratio from the simulated samples when compared to the detected mutation ratio only from the tumour reference material. There are many similarities between DNA extracted by the CEWB method and controls. What stands out is the promising purity of DNA extracted by the CEWB method.

### 3.3 A parallel cohort study to characterise the utility of CEWB for NIPT

Of the 304 samples, 302 were successfully used to construct a library (99.34%). The remaining two samples were discarded. Figure S2 shows the DNA peaks plot of sequencing library PCR product. It shows that we have obtained the cfDNA fragment correctly and that the sequencing library was constructed without gDNA contamination.

Table 1 summarises the NIPT results from a parallel cohort study, with details listed in Table S6. The NIPT results of the treatment group (extracted by CEWB) showed a sensitivity, specificity, positive predictive value, and negative predictive value of 100% (19/19), 99.65% (282/283), 95%, and 100%, respectively. The NIPT results of the treatment (extracted by CEWB) and control (extracted by magnetic bead‐based method from plasma) groups were essentially identical.

**TABLE 1:** The performance of the clinical test in NIPT^a^ with different extraction methods

Method	Control (extracted by magnetic bead‐based method from plasma)	Treatment (extracted by CEWB^b^)
Total	302	302
True negative	283	283
Test negative	282	282
True positive (*n* = 20)	T21/T18/T13:10 + 7 + 2 = 19^c^	T21/T18/T13:10 + 7 + 2 = 19^c^
T21/T18/T13 test positive^c^	T21/T18/T13:10 + 7 + 3 = 20^c^	T21/T18/T13:10 + 7 + 3 = 20^c^
Sensitivity	19/19 = 100%; 95% CI (83.19–100)	19/19 = 100%; 95% CI (83.19–100)
Specificity	282/283 = 99.65%; 95% CI (98.03–99.94)	282/283 = 99.65%; 95% CI (98.03–99.94)
PPV^d^	19/20 = 95%; 95% CI (76.39–99.11)	19/20 = 95%; 95% CI (76.39–99.11)
NPV^e^	282/282 = 100%; 95% CI 98.66–100)	282/282 = 100%; 95% CI 98.66–100)

^a^ non‐invasive prenatal testing.

^b^ cell‐free DNA (cfDNA) extraction from whole blood samples.

^c^ trisomy.

^d^ positive predictive value.

^e^ negative predictive value.

## 4 DISCUSSION

There have been continual demands for automation of experimental operations in molecular laboratories, especially in clinical testing centres that use mature testing technology. For laboratory‐developed tests using NGS, automation in library construction, sequencing, bioinformatics, and report management has been gradually realised.^19^, ^20^, ^21^ However, there has been a continuous demand for automation in sample processing. Automated sample processing systems are complicated, expensive, and not suitable for application in small and medium‐throughput laboratories. Therefore, we proposed CEWB without pre‐processing separation of serum or plasma from WB samples and aimed to create a cost‐effective, simple, small, all‐in‐one machine that combines CEWB with downstream systems. The current automated cfDNA extraction process takes approximately 3 h to complete. This includes 1.5 h for plasma isolation from 96 samples and 1.5 h for automated magnetic bead‐based cfDNA extraction. The automation of CEWB reduces the overall extraction time to 1.5–2 h using the same workstation.

The DNA extracted using CEWB is inferior in quality to that extracted using the Qiagen kit, probably due to the intrinsic nature of these two extraction methods, CEWB being magnetic bead‐based and the Qiagen kit being column‐based. This is consistent with previous studies that have demonstrated that the column‐based method produces more DNA weight yield than the magnetic bead‐based method.^22^ This study did not detect any evidence of contamination from gDNA (Figure S1), potentially explained by the Cell storage solution solidifying the cell membrane and preventing release of gDNA from the cells and by avoiding the protease digestion process.

CEWB was successfully used for NIPT in 302 clinical samples, with excellent sensitivity and specificity, confirming its utility for NIPT. One negative sample confirmed by CMA tested positive for T13 using NIPT, which could be explained, in part, by the interference of the gain of DNA on chromosome 13 (<1 Mb). For this sample, we repeated NIPT three more times all with the same results. As the detection sample size increases, the performance indicators of this method should be reduced to a level consistent with the advertised NIPT product. Notably, since the ratio of foetal DNA to maternal DNA in the WB from pregnant women at 12–24 gestational weeks is usually not less than 3.5%, it is still detectable by CEWB, even if some cfDNA is lost. Extraction of cfDNA from blood or plasma for NIPT is currently the standard method in use, but the centrifugation and transfer of supernatant required by this method is cumbersome, difficult to automate, time‐consuming, and costly for handling large number of samples. However, the CEWB method allows the extraction of cfDNA directly from blood, without the need for a long centrifugation and fractionation process to separate out the plasma, simply by adding the blood to magnetic beads for cfDNA extraction, thus, reducing the overall extraction time to 1.5–2 h per sample and accelerating the construction of a sample processing system. This study did not evaluate the detection limit, so there is a need for further optimisation experiments and more application scenarios where the proportion of tumour cfDNA and exogenous cfDNA is very low, such as diagnosis at stage I of solid tumour or screening for cancer in high‐risk populations. Fragment size distributions from foetal cfDNA or tumour cfDNA are different from those of maternal cfDNA or normal cfDNA.^23^, ^24^, ^25^, ^26^ CEWB can investigate the enrichment and selection of smaller fragment sizes by adjusting the amount and proportion of magnetic beads to increase the proportion and amplify the signal of foetal cfDNA, tumour cfDNA, and exogenous cfDNA in the body.^27^, ^28^ We found that the use of supernatant blood instead of WB extracted by CEWB can effectively increase the cfDNA yield (Table S7). This could be explained by blood cells gradually settling down, retaining only plasma and a small number of blood cells in the supernatant if the peripheral blood is left standing for hours.

Our results have many practical applications. In principle, CEWB can be applied to cfDNA research in nucleic acid molecular diagnostics. In addition to blood, tumour cfDNA has been found in spinal fluid, urine, saliva, and faeces.^29^, ^30^, ^31^, ^32^ The amino magnetic beads of CEWB could directly bind and separate cfDNA from liquids and therefore, it can be used for spinal fluid, urine, saliva, and faecal samples where cfDNA may be directly extracted without the time‐consuming high‐speed centrifugation step. This technology also has the potential to be used to automate sample processing systems combined with sample scanning and coding function modules. The ideal end result is an automatic all‐in‐one machine that combines all processes involved in cfDNA extraction from peripheral blood.

## 5 CONCLUSION

We proposed a cfDNA extraction method from WB samples, termed as CEWB, and demonstrated that it achieves 4.34 ± 0.41 ng/ml plasma DNA yield with promising purity without contamination from gDNA. Subsequent clinical experiments indicated its utility for NIPT. This approach can be applied to non‐invasive liquid biopsy in nucleic acid molecular diagnostics and in automated sample processing systems to eventually realise the goal of an automatic all‐in‐one machine in a cost‐effective, simple, and compact package.

## CONFLICT OF INTEREST

The authors declare no conflict of interest.

## Supporting information

Supplementary Information S1

Supplementary Information S2

Supplementary Information S3

## Data Availability

The data that support the findings of this study are available from the corresponding author upon reasonable request.
