# Supplementary figures and images for: A novel method for extracting circulating cell‐free DNA from whole blood samples and its utility in the non‐invasive prenatal test
Source: Prenat Diagn. 2022 Aug 5;42(9):1173–81. doi: 10.1002/pd.6212 (PMC9541415; doi:10.1002/pd.6212)

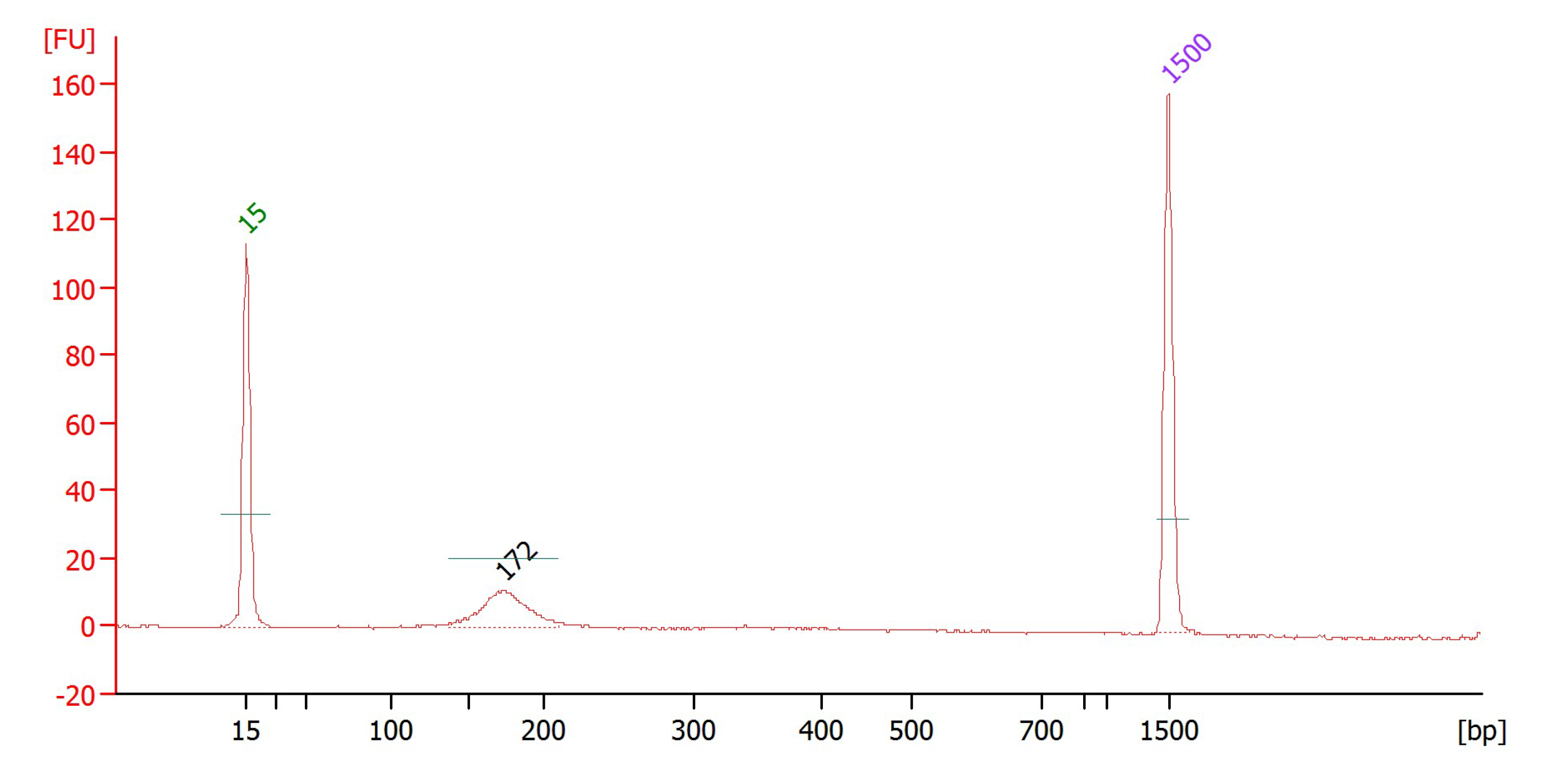

Supplement: Supplementary file 2 — Supplementary Information S2 [file PD-42-1173-s002.tif]

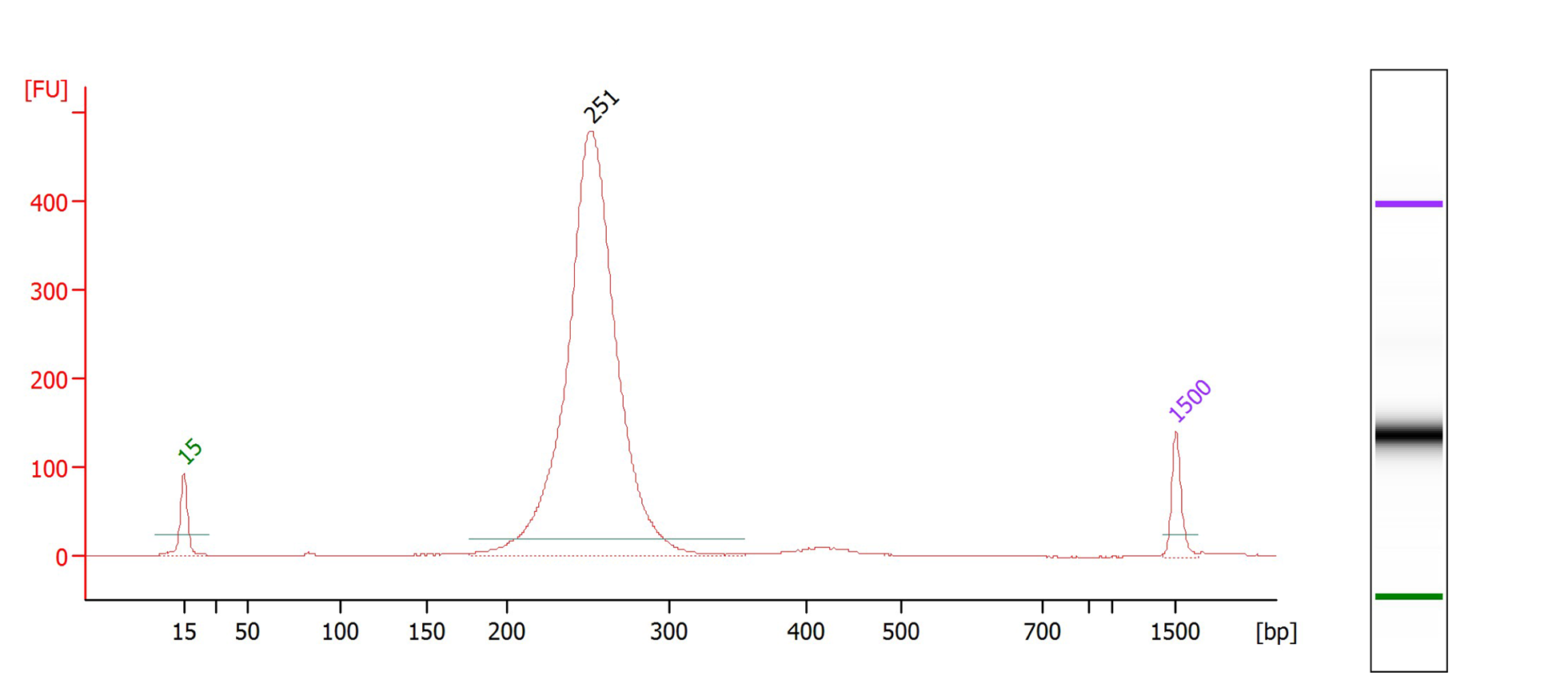

Supplement: Supplementary file 3 — Supplementary Information S3 [file PD-42-1173-s003.tif]
